# Alpha-Gal on the Protein Surface Hampers Transcytosis through the Caco-2 Monolayer

**DOI:** 10.3390/ijms21165742

**Published:** 2020-08-11

**Authors:** Maja Krstić Ristivojević, Jeanette Grundström, Danijela Apostolović, Mirjana Radomirović, Vesna Jovanović, Vlad Radoi, M. B. Gea Kiewiet, Vladana Vukojević, Tanja Ćirković Veličković, Marianne van Hage

**Affiliations:** 1 Department of Medicine Solna, Division of Immunology and Allergy, Karolinska Institutet and Karolinska University Hospital, 17177 Stockholm, Sweden; krstic_maja@chem.bg.ac.rs (M.K.R.); jeanette.grundstrom@ki.se (J.G.); danijela.apostolovic@ki.se (D.A.); gea.kiewiet@ki.se (M.B.G.K.); 2 Faculty of Chemistry, University of Belgrade, 11000 Belgrade, Serbia; radomirovicmirjana@chem.bg.ac.rs (M.R.); vjovanovic@chem.bg.ac.rs (V.J.); 3 Department of Clinical Neuroscience, Center for Molecular Medicine (CMM), Karolinska Institutet, 17176 Stockholm, Sweden; vlad.radoi@ki.se (V.R.); vladana.vukojevic@ki.se (V.V.); 4 Ghent University Global Campus, Yeonsu-gu 21985, Incheon, Korea; 5 Faculty of Bioscience Engineering, Ghent University, 9000 Ghent, Belgium; 6 Serbian Academy of Sciences and Arts, 11000 Belgrade, Serbia

**Keywords:** α-Gal, transcytosis, glycoprotein, glycans, Caco-2 cells, mammalian meat allergy

## Abstract

Transepithelial transport of proteins is an important step in the immune response to food allergens. Mammalian meat allergy is characterized by an IgE response against the carbohydrate moiety galactosyl-α-1,3-galactose (α-Gal) present on mammalian glycoproteins and glycolipids, which causes severe allergic reactions several hours after red meat consumption. The delayed reaction may be related to the processing of α-Gal carrying proteins in the gastrointestinal tract. The aim of this study was to investigate how protein glycosylation by α-Gal affects the susceptibility to gastric digestion and transport through the Caco-2 cell monolayer. We found that α-Gal glycosylation altered protein susceptibility to gastric digestion, where large protein fragments bearing the α-Gal epitope remained for up to 2 h of digestion. Furthermore, α-Gal glycosylation of the protein hampered transcytosis of the protein through the Caco-2 monolayer. α-Gal epitope on the intact protein could be detected in the endosomal fraction obtained by differential centrifugation of Caco-2 cell lysates. Furthermore, the level of galectin-3 in Caco-2 cells was not affected by the presence of α-Gal glycosylated BSA (bovine serum albumin) (BSA-α-Gal). Taken together, our data add new knowledge and shed light on the digestion and transport of α-Gal glycosylated proteins.

## 1. Introduction

Although most glycan antigens do not cause allergic symptoms, during the last decade, the mammalian carbohydrate epitope galactosyl-α-1,3-galactose (α-Gal) has been shown to cause severe allergic reactions [1]. The α-Gal epitope is expressed on non-primate mammalian proteins [2], and patients with mammalian meat allergy have IgE antibodies to α-Gal and report symptoms of anaphylaxis, angioedema, or urticaria 3 to 6 h after red meat consumption [3,4,5]. The timing of the symptoms has been confirmed in food challenges where the patients’ basophils were activated in the same timeframe [6]. To date, it still remains unclear why the symptoms are delayed, and a better understanding of the underlying mechanisms is needed. There is a lack of literature dealing with the fate of α-Gal carrying proteins in the gastrointestinal tract, including processing, uptake and transport of α-Gal carrying proteins by the intestinal epithelium.

Enterocytes of the intestinal epithelium are the most important cells for absorbing nutrients from food and at the same time excluding antigenic macromolecules and microbes from crossing the barrier. The human colon carcinoma cell-line Caco-2 is used as a model system of the intestinal epithelium. When cultured on permeable supports, the cells differentiate into a monolayer that is similar to the small intestinal villus epithelium and this monolayer expresses brush border proteins, tight junctions between cells, and is highly polarized [7,8]. The Caco-2 cell monolayer has been used for investigation of transport of food allergens [9,10,11,12]. In addition, the Caco-2 in vitro model system has been applied to show that allergens can be resistant to digestion and maintain their allergenicity after passing the epithelial barrier [12]. 

Pathogen-derived glycoconjugates can be sensed by mammalian lectins, which are of importance in both innate and acquired immune responses. Galectin-3, the chimera type galectin, can bind to α-Gal [13,14] and is abundantly expressed by intestinal epithelial cells [15]. Furthermore, galectin-3 is upregulated in myeloid and epithelial cells upon microbial stimulation and has mainly pro-inflammatory properties [16]. The question of if and how α-Gal containing proteins are taken up and transported through epithelial cells is still un-answered. Therefore, the aim of this study was to improve the knowledge regarding the gastrointestinal fate of α-Gal carrying proteins. We investigated the impact of α-Gal glycosylation on protein susceptibility to gastric digestion and whether the α-Gal epitope influences protein transcytosis through the Caco-2 cell monolayer.

## 2. Results

### 2.1. α-Gal Glycosylation of BSA Impacts the Pepsin Digestion Pattern

**Figure 1:**
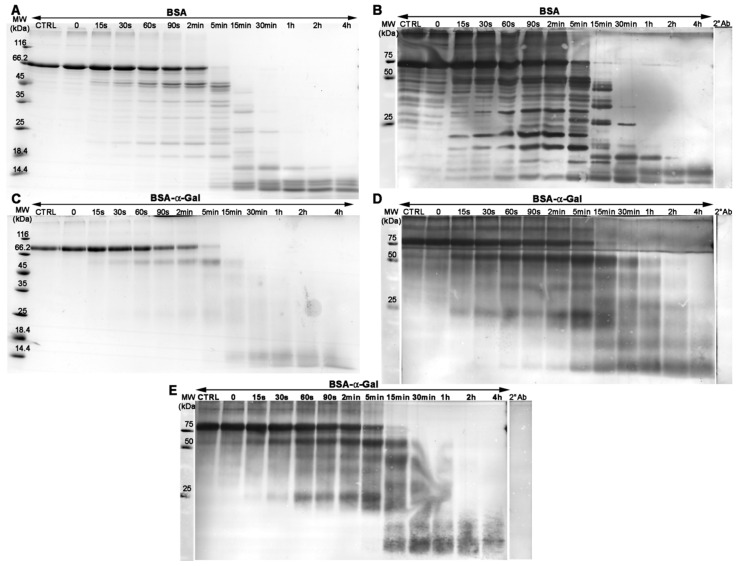
In vitro gastric digestion products of bovine serum albumin (BSA) and α-Gal (galactosyl-α-1,3-galactose) glycosylated BSA (BSA-α-Gal) resolved on sodium dodecyl sulfate polyacrylamide gel electrophoresis (SDS PAGE) and stained by Coomassie Brilliant Blue R-250 (CBB R-250) (**A**,**C**), in vitro gastric digestion products of BSA and BSA-α-Gal analyzed on immunoblot with polyclonal anti-BSA antibody (**B**,**D**), and in vitro gastric digestion products of BSA-α-Gal analyzed on immunoblot with monoclonal anti-α-Gal antibody M86 (**E**). 2°Ab = control of unspecific binding of secondary antibody, MW = Molecular weight markers, and CTRL = undigested protein.

In food allergy, the resistance to pepsin digestion is seen as one of the markers of potential allergenicity. To investigate the effect of α-Gal glycosylation on resistance to pepsin digestion, we compared the pepsin digestion profiles of bovine serum albumin (BSA) carrying α-Gal or N-acetyllactosamine (NAl), and BSA without glycosylation. The digestion products of the different proteins were analyzed by sodium dodecyl sulfate polyacrylamide gel electrophoresis (SDS PAGE) under reducing conditions (Figure 1A,C) and immunoblot using polyclonal anti-BSA IgG antibody. Intact α-Gal glycosylated BSA (BSA-α-Gal) and BSA were detected for up to 15 min. The degradation pattern of BSA-α-Gal was clearly different from that of BSA (Figure 1B,D, respectively). However, both BSA-α-Gal and BSA degraded quickly, where peptides of about 45 kDa for BSA and 55 kDa for BSA-α-Gal appeared already after 15 s of digestion, and became more intense when digestion was prolonged up to 5 min. In addition, digestion products between 25 and 55 kDa were present for up to 2 h for BSA-α-Gal, whereas similar sized products for BSA were only detected up to 30 min. The presence of the α-Gal epitope on the BSA-α-Gal peptides of the molecular weight below 25 kDa is the probable reason for the obtained smeared bands whereas BSA peptides below 25 kDa were distinct. Thus, it seems that α-Gal conjugation of BSA alters the protein susceptibility to pepsinolysis.

Interestingly, NAl, a carbohydrate modification similar in size to α-Gal, conjugated to BSA (BSA-NAl) also inhibited the pepsinolysis susceptibility of the protein. The intact protein was present for up to 2 h of in vitro digestion, while peptides of molecular weights ≥25 kDa were present throughout the entire digestion period (Figure S1).

The α-Gal-content of the peptides obtained during in vitro digestion of BSA-α-Gal was visualized using immunoblot probed with monoclonal anti-α-Gal IgM antibody (Figure 1E). Interestingly, large α-Gal carrying digested peptide fragments, with a molecular weight ≥25 kDa were still, although barely, detectable after 2 h of pepsin digestion.

### 2.2. Fewer Proteins with Conjugated α-Gal Are Transported through the Caco-2 Monolayer

**Figure 2:**
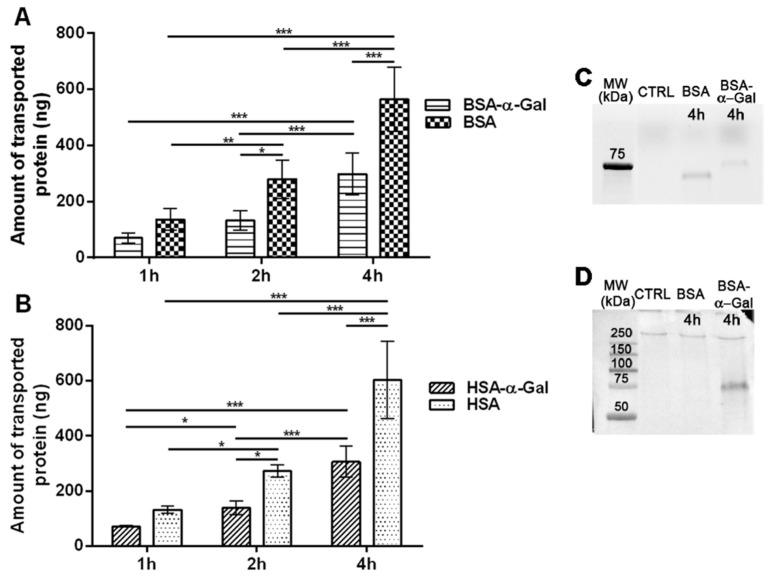
Transport of proteins carrying α-Gal through the intestinal Caco-2 monolayer. (**A**) Time dependent transcytosis of BSA-α-Gal and BSA, *n* = 4, and (**B**) human serum albumin (HSA) carrying α-Gal (HSA-α-Gal) and HSA, *n* = 4. * *p* < 0.05, ** *p* ˂ 0.01 and *** *p* < 0.001 analyzed by two-way ANOVA with Bonferroni’s post hoc test; (**C**) fluorescent detection of BSA-α-Gal and BSA in Caco-2 lysates resolved on sodium dodecyl sulfate polyacrylamide gel electrophoresis (SDS PAGE) gel; (**D**) immunoblot detection of the α-Gal epitope in lysates from Caco-2. MW = Molecular weight markers and CTRL = untreated Caco-2 cells.

In order to monitor the transport of glycosylated proteins over the intestinal epithelium, we established an in vitro model system based on Caco-2 cells. We found that the protein transport rate was constant over time (Table 1). The amount of transported protein after 1 h of incubation with 400 µg of fluorescently labeled BSA-α-Gal was 70 ± 19 ng and after 4 h, the amount had increased to 299 ± 75 ng. When BSA was administered in the same amount, 136 ± 39 ng had been transported after 1 h and 565 ± 113 ng after 4 h of incubation (Figure 2A), approximately 2 times more compared with BSA-α-Gal. In comparison, the transport of human serum albumin (HSA) carrying α-Gal (HSA-α-Gal) was similar to that of BSA-α-Gal and significantly lower compared to the transport of HSA. The amount of transported HSA-α-Gal when 400 µg was applied on the monolayer was 72 ± 3 ng after 1 h of incubation and 306 ± 57 ng of protein after 4 h of incubation, whereas 132 ± 13 ng of HSA had been transported after 1 h of incubation and 603 ± 141 ng after 4 h of incubation (Figure 2B).

Moreover, intact BSA-α-Gal and BSA were detected in Caco-2 cell lysates after 4 h of incubation (Figure 2C). Western blot analysis with chicken single-chain variable fragment (ScFV) anti-α-Gal, as an additional confirmation of the detection of the α-Gal epitope, confirmed the presence of the α-Gal epitope on the BSA-α-Gal (Figure 2D).

### 2.3. The Type of Carbohydrate Moiety Affects Protein Transport through the Caco-2 Monolayer

**Figure 3:**
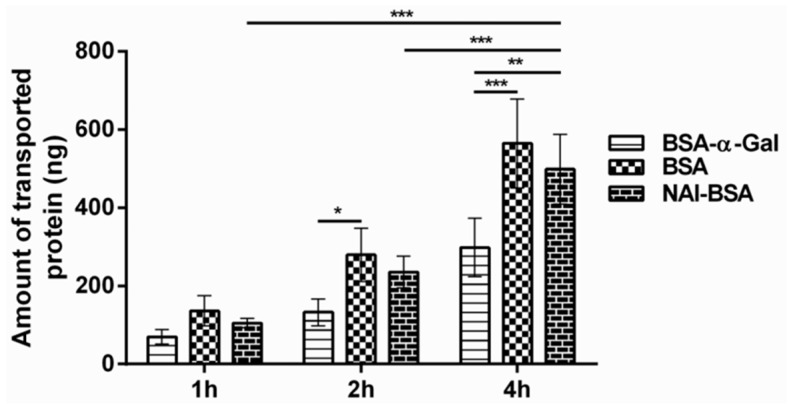
Comparison of transcytosis of BSA-α-Gal, BSA, and N-acetyllactosamine (NAl) conjugated to BSA (BSA-NAl) through the Caco-2 monolayer, *n* = 4, * *p* < 0.05, ** *p* ˂ 0.01 and *** *p* < 0.001 analyzed by two-way ANOVA with Bonferroni’s post hoc test.

To investigate whether transport through the Caco-2 monolayer depends on the type of glycosylation present on the protein, transcytosis of BSA-NAl was monitored at different time points. The amount of transported BSA-NAl when 400 µg was applied on the apical side of the cell monolayer was 105 ± 11 ng after 1 h of incubation and 499 ± 167 ng after 4 h of incubation (Figure 3). The transport of BSA-NAl was comparable to that of BSA and significantly higher compared to the transport of BSA-α-Gal after 4 h of incubation.

### 2.4. α-Gal Carrying Proteins Can Be Detected in Endosomes and Do Not Influence the Level of Galectin-3 in Caco-2 Cells

**Figure 4:**
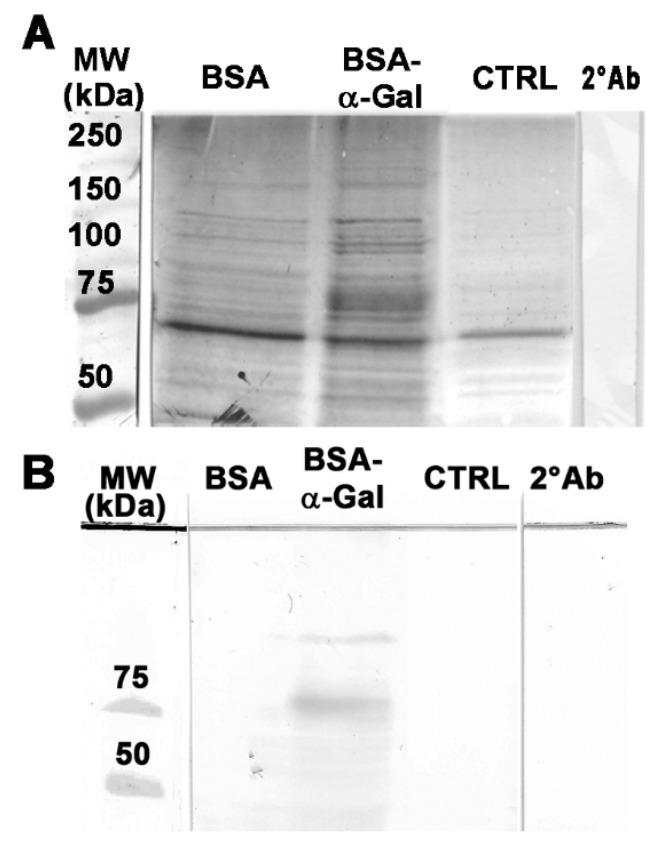
Immunoblot detection of BSA and BSA-α-Gal using (**A**) polyclonal anti-BSA antibody and (**B**) monoclonal anti-α-Gal M86 antibody in the endosomal fraction obtained by differential centrifugation of Caco-2 cell lysates after 4 h of incubation, 2°Ab = control of unspecific binding of secondary antibody, MW = Molecular weight markers, and CTRL = unstimulated Caco-2 cells.

Confocal laser scanning microscopy (CLSM) showed that AF-488 labeled BSA-α-Gal is scattered in the cell cytosol after 4 h of incubation (Figure S2, green). In contrast, no accumulation of labeled BSA was detected, evidenced by the lack of green signal. For further elucidation and confirmation of the spatial distribution of proteins labeled by the fluorescent dye AF-488, differential centrifugation was used to isolate endosomes. Fluorescence detection confirmed the presence of intact proteins in the cell lysates and subsequently in the endosomal fraction of cell lysates after 4 and 24 h of incubation of Caco-2 cells with BSA and BSA-α-Gal (Figure S3A,B). Both BSA and BSA-α-Gal were detected in the corresponding endosomal fraction of Caco-2 cell lysates after 4 and 24 h of incubation using a polyclonal anti-BSA antibody (Figure 4A and Figure S3C). BSA was found in both unstimulated Caco-2 cells (CTRL) and BSA-α-Gal stimulated cells, probably due to its presence in fetal bovine serum (FBS) supplement in the culturing medium used prior to the transport experiment. Importantly, the α-Gal epitope was detected in the endosomal fraction of Caco-2 cell lysates after 4 h of incubation with BSA-α-Gal using the monoclonal anti-α-Gal M86 antibody (Figure 4B). In addition to intact BSA-α-Gal, a band containing the α-Gal epitope corresponding to the molecular weight of 150 kDa in SDS PAGE was detected. Furthermore, the galectin-3 level did not change during transport and uptake of BSA-α-Gal by Caco-2 monolayers (Figure S4).

## 3. Discussion

Most allergic reactions to food are directed against protein epitopes and occur rapidly after ingestion of the allergen. This is in contrast to mammalian meat allergy where the allergic reactions are directed against the carbohydrate α-Gal and occur several hours after intake. We have previously shown that the α-Gal epitope is commonly present in beef proteins and that the allergenicity of red meat proteins is preserved even after cooking [17].

In the present study, we show that protein glycosylation, one of the most common posttranslational modifications, has an impact on digestion stability and, moreover, on the pattern of the resulting peptides. Interestingly, α-Gal glycosylation increased the resistance of BSA to pepsinolysis, and NAl glycosylation hampered pepsinolysis even more. Intact BSA-α-Gal could be detected for up to 15 min of in vitro pepsin digestion similar to BSA, whereas intact BSA-NAl could be detected for up to 2 h. The major difference between BSA-α-Gal and BSA was seen in the digestion patterns, where large digestion products of BSA-α-Gal ≥25 kDa were present for up to 2 h, which was not seen for BSA; in the lower molecular weight region where BSA-α-Gal gave rise to weak peptide bands, BSA generated well-defined peptide bands. Immunoblot analysis of the α-Gal content of the digestion products of BSA-α-Gal confirmed the presence of the α-Gal epitope on large peptides even after 2 h of in vitro pepsin digestion. Of importance, the major peptides carrying the α-Gal epitope were detected at molecular weights between 25 and 55 kDa, although faintly stained, implicating a prolonged survival of proteins during gastric digestion and a prolonged availability for uptake by intestinal cells. Moreover, we have previously shown that α-Gal containing peptides resulting from pepsinolysis are allergenic, reinforcing their role as clinically relevant food allergens [18]. These findings are in accordance with a previous study that demonstrated that glycosylation protects the egg allergen ovomucoid from pepsinolysis [19]. 

Since food allergens are transported through gut epithelial cells, it is of importance to investigate transcytosis of novel allergenic foods. We demonstrate that the presence of the α-Gal epitope on BSA hampers transport through the intestinal Caco-2 monolayer compared to unconjugated BSA, which could be a possible reason why the allergic reaction to α-Gal is delayed. Interestingly, the transport of BSA-NAl was higher than that of BSA-α-Gal, even though the carbohydrates α-Gal and NAl are of similar size, suggesting that the slower transport is glycosylation specific. In contrast to our results, Roman-Carrasco et al. [20] showed that only α-Gal bound to lipids, not to proteins, could be detected in the basolateral medium after transport through the Caco-2 monolayer. However, different methods for the detection of transported proteins and peptides were used and the amount of α-Gal containing proteins added was different. We applied 800 µg/mL of pure α-Gal containing protein compared to 1 mg/mL of digested red meat extract, suggesting that the lower amount of α-Gal containing proteins in the extract could not be detected.

It has been shown that mannosylated BSA is transported by endosomes and lysosomes in alveolar macrophages [21]. BSA-α-Gal and the α-Gal epitope on the intact protein were detected in the endosomal fraction of Caco-2 cell lysates, and intact protein was detected even after 24 h of incubation. One of the possible explanations of this delayed processing is that the presence of α-Gal epitopes on the protein surface leads to steric interference that impedes access to cellular and endosomal proteases involved in protein degradation. It has been demonstrated that protein modifications, e.g., lactosylation, can influence protein hydrolysis catalyzed by cathepsin D, a protease present in endolysosomal cell compartments [22]. 

In this model of intestinal epithelium, we could not see any effect on the expression of galectin-3 after stimulation with BSA-α-Gal. BSA-α-Gal is a harmless protein and since healthy gut epithelium should be tolerant to commensal bacteria of the gut flora, which expresses α-Gal, an inflammatory response cannot be expected.

In conclusion, gastric digestion of BSA-α-Gal results in a rich spectrum of peptides abundant in α-Gal epitopes. Moreover, α-Gal carrying proteins are transported through the intestinal Caco-2 monolayer in significantly lower amounts compared to non α-Gal carrying proteins. BSA-α-Gal is notably accumulated and retained in endosomes of the intestinal cells. These findings contribute to the understanding of the mechanisms underlying the delayed onset of mammalian meat allergic symptoms. However, further research is needed in order to elucidate the kinetics of the processing of proteins containing the α-Gal epitope.

## 4. Materials and Methods 

### 4.1. Reagents 

BSA and HSA were obtained from Sigma-Aldrich (Merck, Steinheim, Germany). BSA-α-Gal, HSA-α-Gal, and BSA-NAl were obtained from Dextra Laboratories (Reading, UK).

### 4.2. Protein Sample Preparation

Proteins were conjugated to AlexaFluor488 (AF488) fluorescent dye using an AF488 protein labeling kit (Invitrogen, Molecular Probes, Inc., Eugene, OR, USA) according to the manufacturer’s instructions. Protein concentration was determined with a Pierce Bicinchoninic acid (BCA) Protein Assay Kit (Pierce Biotechnology, Rockford, IL, USA).

### 4.3. In Vitro Gastric Digestion

In vitro gastric digestion was performed as previously described [23] with slight modification. Briefly BSA-α-Gal, BSA-NAl, and BSA (AF488 labeled) were pre-incubated at 37 °C for 15 min, before pepsin (Sigma-Aldrich) in simulated gastric fluid (SGF) was added in a 1:3 (volume protein/volume SGF with pepsin) ratio. The final enzyme activity was 8 U pepsin per mg protein. For preparation of 4× SGF with pepsin, see Supplementary Table S1. The pH of the digestion mixture was adjusted to 3 by the addition of 1 M HCl. Simulated gastric digestion was monitored for up to 4 h at 37 °C, and aliquots were withdrawn from a single digestion mixture at different time points for further analysis. The digestion was stopped by adding 1 M NaHCO_3_, raising the pH to 7.5. Pepsin control is shown in Figure S5.

### 4.4. SDS PAGE Analysis 

Digestion products and intracellular proteins were analyzed by SDS PAGE according to Laemmli [24] under reducing conditions. The analysis was carried out using a Hoefer Scientific Instruments apparatus (Holliston, MA, USA) with a discontinuous buffer system, 14% polyacrylamide (PAA) hand-cast gels for digestion products, and with pre-cast any kDa PAA gels (Bio-Rad, Hercules, CA, USA) for intracellular proteins. For digestion products, resolved PAA gels were stained using Coomassie Brilliant Blue R-250 (Sigma-Aldrich) and scanned using the Typhoon FLA 7000 laser scanner. Intracellular proteins were detected on a ChemiDoc system (Bio-Rad).

### 4.5. Immunoblot for Detection of the α-Gal Epitope, BSA, and Galectin-3

Immunoblots for detection of α-Gal in BSA-α-Gal digests, the intestinal Caco-2 cell monolayer, and endosomal fraction lysates were performed. Proteins resolved by SDS PAGE were transferred to polyvinylidene difluoride (PVDF) membranes (0.2 µm pore size) using an EBU-4000 Semi Dry Blotting System (Fisher Scientific Company L.L.C., Pittsburgh, Pennsylvania, USA) or a Bio-Rad turbo system. The membranes were probed with monoclonal anti-α-Gal antibody M86 (Enzo Life Sciences, Farmingdale, NY, USA) diluted 1:5 in 0.1% HSA in tris-buffered saline—Tween 20 (TBS-T) for 3 h followed by alkaline phosphatase (AP)-labeled goat anti-mouse IgM antibody (Southern Biotech, Birmingham, AL, USA) diluted 1:3000 in TBS-T for 1 h at room temperature (RT). Alternatively, the membranes were incubated for 2 h at RT with the chicken ScFV anti-α-Gal [25] (ScFV, a kind gift from the Glycoscience group at the National University of Ireland, Galway, Ireland) diluted 1:7500 in 0.2% HSA in phosphate buffered saline—Tween 20 (PBS-T). Subsequently, the membrane was incubated with 1 µg/mL monoclonal mouse-anti-HA antibody (H3663, Sigma-Aldrich) in 0.2% HSA in PBS-T for 1 h at RT followed by goat-anti-mouse IgG conjugated to AP (Jackson Immunoresearch, West Grove, PA, USA) diluted 1:1000 in 0.2% HSA in PBS-T for detection. Reactive bands were visualized as stated above on the ChemiDoc system. For immunodetection of in vitro pepsin digestion, peptides of BSA-α-Gal, BSA-NAl, and BSA were resolved by SDS PAGE and transferred to PVDF membranes. The membranes were probed with rabbit polyclonal anti-BSA IgG antibody (Invitrogen, A11133) diluted 1:1000 in 0.1% HSA for 1.5 h at RT followed by goat anti-rabbit IgG conjugated to AP (ab97072, Abcam, Cambridge, MA, USA) diluted 1:5000 in 0.1% HSA for 1 h at RT. Reactive bands were visualized as stated above. Immunoblot detection of galectin-3 in Caco-2 cell monolayer lysates after 4 and 24 h of incubation with BSA-α-Gal was performed using goat polyclonal anti-human galectin-3 IgG antibody (R&D systems, Abingdon, UK, AF1154) diluted 1:10,000 in 0.1% BSA for 1 h at RT followed by rabbit anti-goat IgG conjugated to AP (Sigma-Aldrich, A4187) diluted 1:20,000 in 0.1% BSA for 1 h at RT. Immunoreactive bands were visualized using nitro blue tetrazolium (NBT) and 5-bromo-4-chloro-3-indolyl phosphate (BCIP) substrates (Bio-Rad). 

### 4.6. Transport through the Caco-2 Monolayer

For transport studies, the Caco-2 (HTB37) cell line was obtained from The American Type Culture Collection (ATCC, Manassas, VA, USA). Caco-2 cells were cultured in Minimum Essential medium (MEM) supplemented with 10% of FBS, 2 mM glutamine, 1% of nonessential amino acids (NEAA), 100 IU/mL penicillin, 0.1 mg/mL streptomycin, and 50 µg/mL gentamicin (all from Sigma-Aldrich). Cells were incubated at 37 °C in a humidified atmosphere with 5% CO_2_. Cells at passages of 23–68 were seeded at 6 × 10^4^ cells/cm^2^ [26] on cell culture inserts for 12-well plates (ThinCert, Greiner Bio-One GmbH, Frickenhausen, Germany) and cultured for 21 days before the start of the experiments. Medium was replaced three times per week. Prior to the transport studies and at the end of experiment, transepithelial electrical resistance (TEER) was measured using a Millicell-ERS VoltOhmmeter (Millipore, Amsterdam, Netherlands). Only cell monolayers with TEER > 300 Ωcm^2^ were used, and none of the added proteins had any effect on the TEER. Transport studies were started by adding 400 µg of AF488-labeled proteins in 500 µL (0.8 mg/mL) complete MEM (cMEM) to the apical compartment. Aliquots were withdrawn from the basolateral compartment after 1, 2, and 4 h of incubation and the fluorescence was measured using a FluoroMax-4 spectrofluorometer (Horiba Scientific, Kyoto, Japan). Protein concentration was calculated from standard curves of the corresponding protein, with the limit of detection (LOD) of 0.011 µg/mL (LOD = 3S_a_/b, where S_a_ is the standard deviation of the response and b is the slope of the calibration curve).

### 4.7. Intracellular Visualization of Proteins and Isolation of the Endosomal Fraction of the Caco-2 Cell Monolayer

To visualize internalization of AF488-labeled proteins, CLSM was employed [27]. The detailed protocol is described in the Supplementary Information. Isolation of the endosomal fraction of Caco-2 cell lysates was performed as previously described [28] with slight modification. Briefly, Caco-2 cells were cultured in the same conditions as for transport studies in 6-well plates. Before addition of treatments, the monolayers were washed 3 times with PBS and cultivating medium was replaced with medium without FBS. Caco-2 cell monolayers were incubated with 100 µg of AF488-labeled BSA or BSA-α-Gal for 4 and 24 h, unstimulated cells were used as control. After incubation, the cells were washed 3 times with PBS and detached from the culture inserts using ice-cold PBS-EDTA solution. The cell pellets were washed 3 times with PBS and lysed with lysis buffer (250 mM sucrose, 50 mM Tris-Cl (pH 7.4), 5 mM MgCl_2_, 1 mM EDTA, 1 mM egtazic acid (EGTA)) with a protease inhibitor cocktail (Sigma-Aldrich). The suspended cell lysates were left overnight at −80 °C. The next day, endosomes were harvested in the third pellet after three centrifugation steps of the supernatant: 1st (1000× *g*, 10 min, performed twice and the supernatants were pooled), 2nd (16,000× *g*, 20 min), and 3rd (100,000× *g*, 1 h) for immunoblot analysis. All centrifugation steps were performed at 4 °C, and for the last ultracentrifugation step a Sorvall WX80 ultracentrifuge (Thermo Electron LED GmbH, Langenselbold, Germany) with T-880 rotor was used.

### 4.8. Statistical Analysis

Data are presented as mean ± standard error (SD) and were analyzed using GraphPad Prism software version 6.04 software for Windows (La Jolla, CA, USA, www.graphpad.com). Each sample was analyzed in duplicate with two biological replicates. The differences were analyzed by two-way ANOVA. The transport rates were calculated by linear regression. Differences were considered significant if *p* < 0.05.

## Figures and Tables

**Table 1:** Transport rates of the different proteins over the Caco-2 monolayer.

Protein	Transport Rate (ng/h)	95% CI	r^2 †^	*p*-Value ^‡^
BSA	142.8	103.9–181.7	0.87	˂0.001
BSA-α-Gal	77.2	53.1–101.3	0.84	˂0.001
HSA	158	117.2–198.7	0.88	˂0.001
HSA-α-Gal	78.8	61.0–96.6	0.91	˂0.001
BSA-NAl	135.0	83.4–186.6	0.77	˂0.001

^†^ Goodness of fit, ^‡^ if the slope is different from zero.

## Supplementary Materials

Supplementary materials can be found at https://www.mdpi.com/1422-0067/21/16/5742/s1. Figure S1: Immunoblot analysis of in vitro gastric digestion products from BSA-NAl using polyclonal anti-BSA antibody; Figure S2: Uptake of BSA-α-Gal and BSA in Caco-2 cells after 4 h of incubation at 37 °C analyzed by confocal laser scanning microscopy; Figure S3: Fluorescent detection of BSA and BSA-α-Gal; Figure S4: Western blot detection of galectin-3 using polyclonal anti-human galectin-3 antibody in Caco-2 cell lysates; Figure S5: SDS PAGE analysis of pepsin control solution with pepsin only at 0 and 4 h time point, stained by CBB-R; Table S1: Preparation of stock solution of simulated gastric fluid (SGF).
