# Supplementary material for: Alpha-Gal on the Protein Surface Hampers Transcytosis through the Caco-2 Monolayer
Source: Int J Mol Sci. 2020 Aug 11;21(16):5742. doi: 10.3390/ijms21165742 (PMC7461108; doi:10.3390/ijms21165742)
Supplement: Supplementary file 1 [file ijms-21-05742-s001.pdf]

International Journal of *Molecular Sciences*

MDPI

Supplementary

Alpha-Gal on the Protein Surface Hampers Transcytosis Through the Caco-2 Monolayer

Maja Krstić Ristivojević [1,2], Jeanette Grundström [1], Danijela Apostolović [1], Mirjana Radomirović [2], Vesna Jovanović [2], Vlad Radoi [3], M.B. Gea Kiewiet [1], Vladana Vukojević [3], Tanja Ćirković Veličković [2,4,5,6,*,†], Marianne van Hage [1,*,†]

[1] Department of Medicine Solna, Division of Immunology and Allergy, Karolinska Institutet and Karolinska University Hospital, 17177 Stockholm, Sweden; krstic_maja@chem.bg.ac.rs (M.K.R.); jeanette.grundstrom@ki.se (J.G.), danijela.apostolovic@ki.se (D.A.), gea.kiewiet@ki.se (M.B.G.K)
[2] Faculty of Chemistry, University of Belgrade, 11000 Belgrade, Serbia, radomirovicmirjana@chem.bg.ac.rs (M.R.), vjovanovic@chem.bg.ac.rs (V.J.)
[3] Department of Clinical Neuroscience, Center for Molecular Medicine (CMM), Karolinska Institutet, 171 76 Stockholm, Sweden; vlad.radoi@ki.se (V.R.), vladana.vukojevic@ki.se (V.V.)
[4] Ghent University Global Campus, 21985 Yeonsu-gu, Incheon, South Korea
[5] Faculty of Bioscience Engineering, Ghent University, 9000 Ghent, Belgium
[6] Serbian Academy of Sciences and Arts, 11000 Belgrade, Serbia
[*] Correspondence: tcirkov@chem.bg.ac.rs (T.C.V.); marianne.van.hage@ki.se (M.v.H.)
[†] Co-last authors.

Abstract: Transepithelial transport of proteins is an important step in the immune response to food allergens. Mammalian meat allergy is characterized by an IgE response against the carbohydrate moiety galactosyl-α-1,3-galactose (α-Gal) present on mammalian glycoproteins and glycolipids, which causes severe allergic reactions several hours after red meat consumption. The delayed reaction may be related to the processing of α-Gal carrying proteins in the gastrointestinal tract. The aim of this study was to investigate how protein glycosylation by α-Gal affects the susceptibility to gastric digestion and transport through the Caco-2 cell monolayer. We found that α-Gal glycosylation altered protein susceptibility to gastric digestion, where large protein fragments bearing the α-Gal epitope remained for up to 2 h of digestion. Furthermore, α-Gal glycosylation of the protein hampered transcytosis of the protein through the Caco-2 monolayer. α-Gal epitope on the intact protein could be detected in the endosomal fraction obtained by differential centrifugation of Caco-2 cell lysates. Furthermore, the level of galectin-3 in Caco-2 cells was not affected by the presence of α-Gal glycosylated BSA (bovine serum albumin) BSA-α-Gal. Taken together, our data add new knowledge and shed light on the digestion and transport of α-Gal glycosylated proteins.

Keywords: α-Gal; transcytosis; glycoprotein; glycans; Caco-2 cells; mammalian meat allergy

Supplementary Information

1. Materials and Methods

1.1. Intracellular Detection of Proteins

To visualize internalization of AF488 labeled proteins by confocal laser scanning microscopy (CLSM), 90,000 (9.4 × 103 cells/cm²) Caco-2 cells were seeded on sterile 22 × 22 mm coverslips kept in 6-well plates. The cells were cultured for 21 days in complete Minimum Essential Medium (cMEM) that was refreshed three times per week. On day 21, the medium was removed and 100 μg of AF488 conjugated BSA (bovine serum albumin) or BSA-α-Gal in 2 mL of cMEM was added and the cells

were incubated for 4 h at 37 °C. Thereafter, the cells were incubated with 0.01% $CaCl_2$ in Phosphate-buffered saline (PBS) for 5 min and fixed and permeabilized with ice cold methanol at −20 °C for 10 min. The cells were blocked for 1 h at room temperature (RT) with 1% BSA in PBS and immediately incubated with primary rabbit-anti-human-zonula occludens-1 (ZO-1, Life Technologies, Thermo Fischer Scientific) diluted 1:100 in 1% BSA in PBS overnight at 4 °C. The second day, the cells were incubated with secondary goat-anti-rabbit-IgG-Alexa555 (Life Technologies) diluted 1:400 in 1% BSA in PBS for 75 min at RT before mounting with Prolong Gold Antifade reagent (Invitrogen) on to microscope slides.

CLSM was performed as previously described [26] using a LSM 510 Meta system (Carl Zeiss, Jena, Germany) individually modified to allow imaging with improved detection efficiency using avalanche photodiodes. An alpha Plan-Fluar 100×/1.45 oil immersion objective (Zeiss MicroImaging GmbH, Jena, Germany) was used for all images. Images were acquired at 1024 × 1024 pixel resolution, scanning speed 12.6 μs/pixel, without averaging, and prepared for publication using the Zeiss LSM Image Browser software.

Supplementary Table S1. Preparation of stock solution of simulated gastric fluid (SGF). The mass is calculated for a final volume of 200 mL for 4× concentrate SGF.

SGF pH 3		
**Constituent**	**4× SGF***	**Final conc. in SGF**
	g	mmol/L
KCl	0.41	6.90
KH_2PO_4	0.10	0.90
$NaHCO_3$	1.68	25.00
NaCl	2.21	47.20
$MgCl_2(H_2O)_6$	0.02	0.10
$(NH_4)_2CO_3$	0.08	0.50
$CaCl_2(H_2O)_2$	0.02	0.15
	**For pH adjustment**	
	**mL (12M[a])**	**mmol/L**
HCl	1.04	15.6

* to prepare 200 mL of 4× SGF solution.

[a] molarity of stock solution.

2. Results

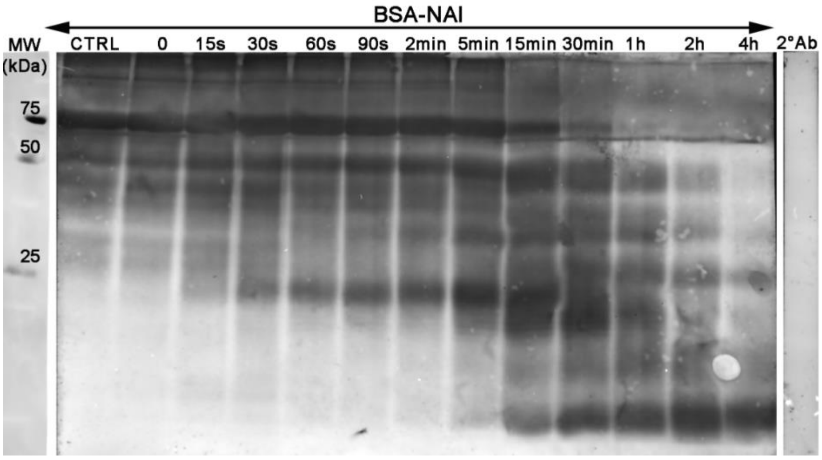

Supplementary Figure S1. Immunoblot analysis of in vitro gastric digestion products from BSA-NAl using polyclonal anti-BSA antibody. 2°Ab = control of unspecific binding of secondary antibody, MW = Molecular weight markers and CTRL = undigested protein.

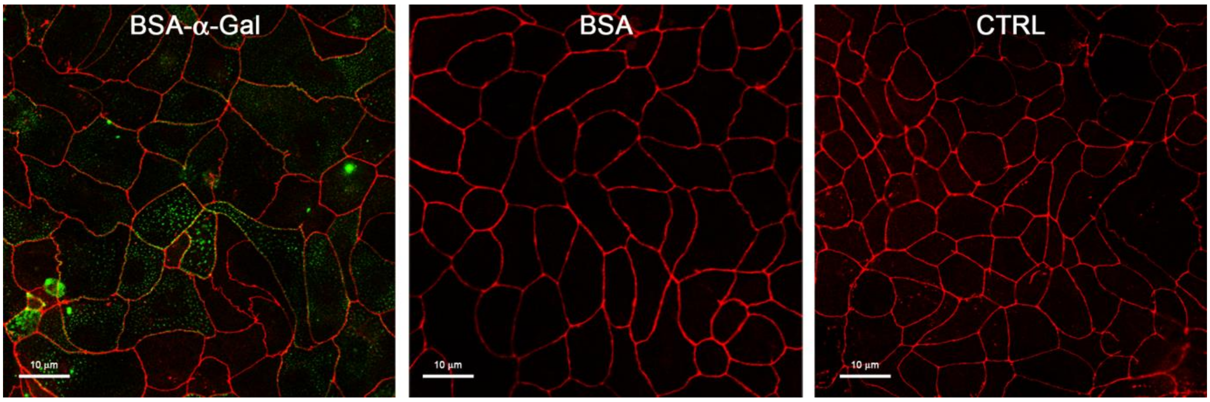

Supplementary Figure S2. Uptake of BSA-α-Gal and BSA in Caco-2 cells, after 4 h of incubation at 37 °C analyzed by confocal laser scanning microscopy. Green = BSA-α-Gal or BSA, red = ZO-1.

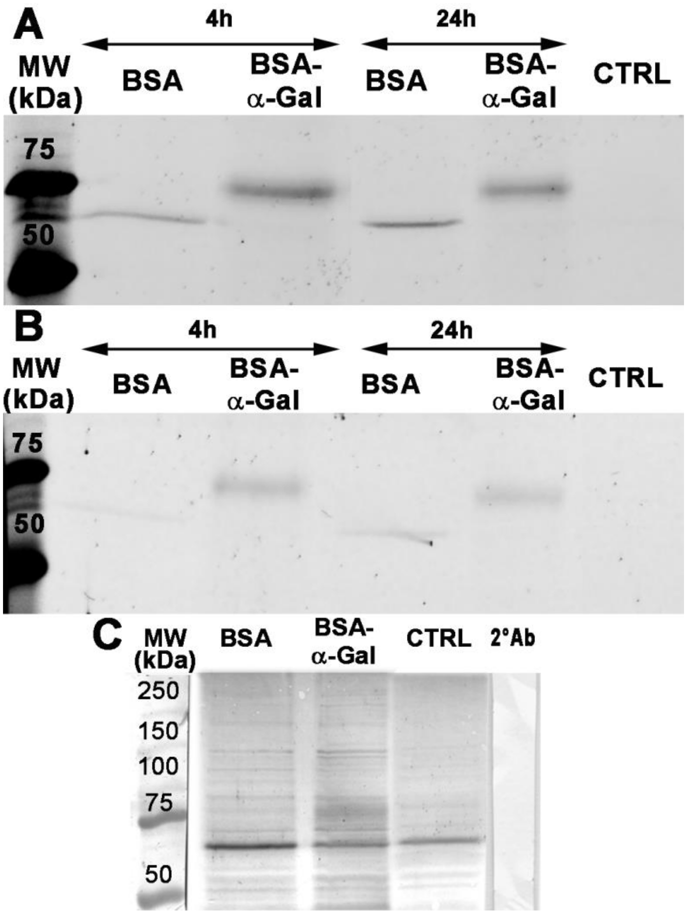

Supplementary Figure S3. Fluorescent detection of BSA and BSA-α-Gal in (**A**) Caco-2 cell lysates and (**B**) endosomal fraction resolved on SDS-PAGE, (**C**) Immunoblot detection of BSA and BSA-α-Gal using polyclonal anti-BSA antibody in the endosomal fraction after 24 h of incubation with the Caco-2 monolayer, 2°Ab = control of unspecific binding of secondary antibody, MW = Molecular weight markers and CTRL = unstimulated Caco-2 cells.

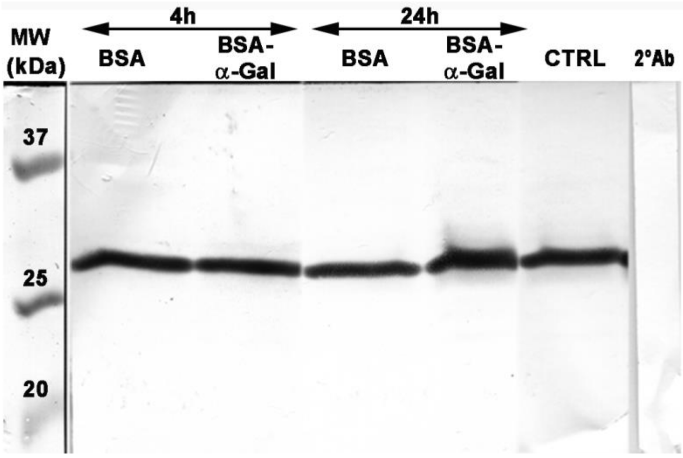

Supplementary Figure S4. Western blot detection of galectin-3 using polyclonal anti-human galectin-3 antibody in Caco-2 cell lysates, 2°Ab = control of unspecific binding of secondary antibody, MW = Molecular weight markers and CTRL = untreated Caco-2 cells lysate.

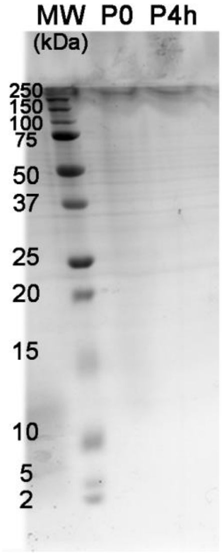

Supplementary Figure S5. SDS-PAGE analysis of pepsin control solution with pepsin only at 0 and 4 h time point, stained by CBB-R. MW = Molecular weight markers.

References

26. Vukojevic, V.; Heidkamp, M.; Ming, Y.; Johansson, B.; Terenius, L.; Rigler, R. Quantitative single-molecule imaging by confocal laser scanning microscopy. *Proc Natl Acad Sci U S A* **2008**, *105*, 18176–18181, doi:10.1073/pnas.0809250105.
